# Time-resolved 3D imaging of two-phase fluid flow inside a steel fuel injector using synchrotron X-ray tomography

**DOI:** 10.1038/s41598-020-65701-x

**Published:** 2020-05-26

**Authors:** Aniket Tekawade, Brandon A. Sforzo, Katarzyna E. Matusik, Kamel Fezzaa, Alan L. Kastengren, Christopher F. Powell

**Affiliations:** 1 Energy Systems Division, Argonne National Laboratory, Lemont, IL USA; 2 X-ray Science Division, Argonne National Laboratory, Lemont, IL USA

**Keywords:** Mechanical engineering, Fluid dynamics, Imaging techniques

## Abstract

The multiphase flow inside a diesel injection nozzle is imaged using synchrotron X-rays from the Advanced Photon Source at Argonne National Laboratory. Through acquisitions performed at several viewing angles and subsequent tomographic reconstruction, *in-situ* 3D visualization is achieved for the first time inside a steel injector at engine-like operating conditions. The morphology of the internal flow reveals strong flow separation and vapor-filled cavities (cavitation), the degree of which correlates with the nozzle’s asymmetric inlet corner profile. Micron-scale surface features, which are artifacts of manufacturing, are shown to influence the morphology of the resulting liquid-gas interface. The data obtained at 0.1 ms time resolution exposes transient flow features and the flow development timescales are shown to be correlated with *in-situ* imaging of the fuel injector’s hydraulically-actuated valve (needle). As more than 98.5% of the X-ray photon flux is attenuated within the steel injector body itself, we are posed with a unique challenge for imaging the flow within. Time-resolved imaging under these low-light conditions is achieved by exploiting both the refractive and absorptive properties of X-ray photons. The data-processing strategy converted these images with a signal-to-noise ratio of ~ 10 into a meaningful dataset for understanding internal flow and cavitation in a nozzle of diameter 200 μm enclosed within 1–2 millimeters of steel.

## Introduction

As fuel injectors today utilize higher injection pressures and smaller nozzle orifices to facilitate efficient spray formation and lower emissions in internal combustion engines, manufacturing deviations from nominal nozzle design have become ever more stringent considerations. Even in larger diesel injectors for heavy duty applications, the orifices diameters can be as small as 200 *μ*m^1^. The variability in their geometric features such as inlet corner sharpness and orifice diameter have been shown to influence internal flow, causing two-phase phenomena such as cavitation or flow-separation near the nozzle walls^2–11^. The occurrence of cavitation, or vapor-filled cavities, can not only lead to erosion in injectors, but also have an impact on the downstream spray development. Given the opaque nature of this flow system, several studies have reported visualization of cavitation using visible-light diagnostics using purpose-built transparent replicas^2,6–8^ and practical diesel injectors that have been modified for optical access^3,9^. In one such notable work, Arcoumanis *et al*. studied a real-size diesel injector with optical access and high magnification to reveal cavitation initiated from the inlet corner as well as string cavitation in the sac^3^.

In contrast to visible light, X-ray diagnostics are not limited by the opacity of a steel injector body as has been demonstrated in previous studies where line-of-sight imaging was conducted inside diesel injector nozzles without any optical access^12,13^. X-ray imaging enables density-field measurements which provide valuable insight into two-phase flows, as demonstrated by Mäkiharju *et al*. in their fundamental study of bubbly cavitating flows behind a backward-facing step^14^. Recently, Moon *et al*. visualized vortex flow in a steel nozzle using synchrotron X-rays^15^. In this study, the fuel was doped with tracer and they obtained 2D line-of-sight images to infer about a flow-field rich in 3D information. Also using synchrotron X-rays, Karathanassis *et al*. first observed vortical cavitation in enlarged nozzles made from carbon-fiber composite material^16^. The transparency of carbon-fiber to X-rays made it possible to achieve high frame rates (347 ns exposure) and observe transient features in the flow. X-ray imaging combined with computed tomography enables the visualization of 3D flow-fields and this has been demonstrated previously on enlarged X-ray-transparent nozzles made from polymer materials^17–19^. In 2012, Bauer *et al*.^19^ built a large cavitation channel (20 mm diameter) and, using a medical CT scanner, reconstructed snapshots of the flow of water through the cross-section, demonstrating the success of this technique in capturing high-speed two-phase flow phenomena. In another study, Mitroglou *et al*.^17^ conducted experiments on fuel flow through a smaller, 3 mm diameter nozzle (also of polymer material), with a voxel resolution of 15 *μ*m. They designed the nozzle to induce flow separation from one side of the inlet cross-section, causing cavitation at even a small pressure drop of 1–4 MPa. They showed asymmetric cavitation with quantifiable measurements of void fraction in the cavitation zone.

**Figure 1:**
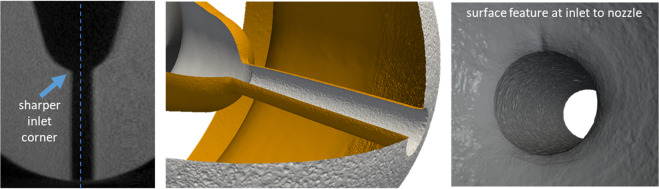
A surface representation derived from high-resolution X-ray computed tomography of the fuel injector (ECN Spray C37) used in this study reveals an asymmetrically drilled hole and a 20 **μ**m wide ridge on the nozzle wall at the inlet. Slice from synchrotron X-ray CT scan (left), full iso-surface (middle), zoom-in view of inlet to nozzle (right). Images produced with data from Matusik *et al*.^1^.

A key limitation of many of previous studies employing 3D X-ray imaging is that the nozzle material, size and operating conditions were not representative of practical injectors, considering typical diesel nozzles are made from steel, have diameters of 100–200 *μ*m, and operate at injection pressures of 100–200 MPa. In 2017, Matusik *et al*.^1^ conducted high-resolution X-ray tomography of diesel injector nozzles openly shared for research purposes within the Engine Combustion Network (ECN)^20^ and showed that walls of the flow orifices were rich in micron-scale surface features. Specifically, they observed that in one such nozzle (“Spray C37”), the orifice was drilled off-center with respect to the sac region (upstream of the orifice). A 20 *μ*m wide ridge was observed at the inlet corner that could perturb the flow as it progressed into the nozzle. This can be seen in Fig. 1. The spatial resolution of this data in terms of minimum resolvable feature size was 1.8 *μ*m (1 *μ*m voxels). Subsequently, Sforzo *et al*. used line-of-sight X-ray imaging to observe persistent cavitation from the sharper inlet corner in the Spray C37 nozzle^13^. They estimated that the resulting cavitation layer could block a significant portion of the flow cross-section, resulting in reduced rate of injection compared to the nominal design with a symmetric inlet corner profile. However, the exact nature of the flow cross-section and the impact of surface features in the nozzle could not be determined from line-of-sight imaging alone, especially given the polychromatic nature of the illumination. This sensitivity to geometry found in the ECN Spray C37 motivated the present work and its choice as the fuel injector of interest.

From an experimental physics standpoint, the aim of the present work was to visualize the 3D flow-field inside a real-size steel diesel injector without any optical access or modifications using a synchrotron X-ray source. Imaging through steel, even using high-flux synchrotron radiation, poses severe limitations on the achievable time-resolution due to the high attenuation coefficient of iron. Furthermore, the flow-field is reconstructed from X-ray projections collected at various angles acquired from several injection events, resulting in time- and ensemble- averaged snapshots which do not capture stochastic phenomena such as string cavitation or shedding/collapse. This is an inherent limitation in any work that uses computed tomography^17–19^. Regardless of these constraints, some of the persistent effects on flow morphology due to surface features and geometric variabilities that occur during manufacturing can still be observed, yielding valuable information.

To obtain the 3D flow-field, the fuel injector, connected to a high-pressure fuel (n-dodecane) supply operating at 150 MPa, was mounted on a rotational stage and inserted into a sealed chamber with X-ray transparent windows. A high-speed camera, synced with the injection event, recorded 2D images of approximately two hundred injection events per viewing angle. Hence, the data were *ensemble-averaged* over several thousand injection events, each lasting 1–3 ms. As such, the images show persistent features of the nozzle flow-field, and will miss features that appear in small numbers of injection events, highly dynamic features such as cavitation cloud shedding, or chaotically moving features such as string cavitation. Images are captured with a resolution of ~ 2 *μ*m/pixel. Each acquisition is performed with a time resolution as short as 100 µs, providing a time-resolved visualization of how the cavitation/flow-separation layer develops over the injection event. Surface features in the orifice are shown to affect the flow morphology. A direct correlation is drawn between the azimuthally-varying inlet corner curvature and the asymmetric nature of the flow separation. As these high-speed phenomena occur behind a millimeter-thick steel wall, we are posed with a challenge – a compromise between photon count and time resolution. The experimental plan and imaging technique developed to tackle this challenge are discussed in the methods section. Parts of this work were previously communicated but not peer-reviewed^21–23^.

## Results

### Needle lift profile

**Figure 2:**
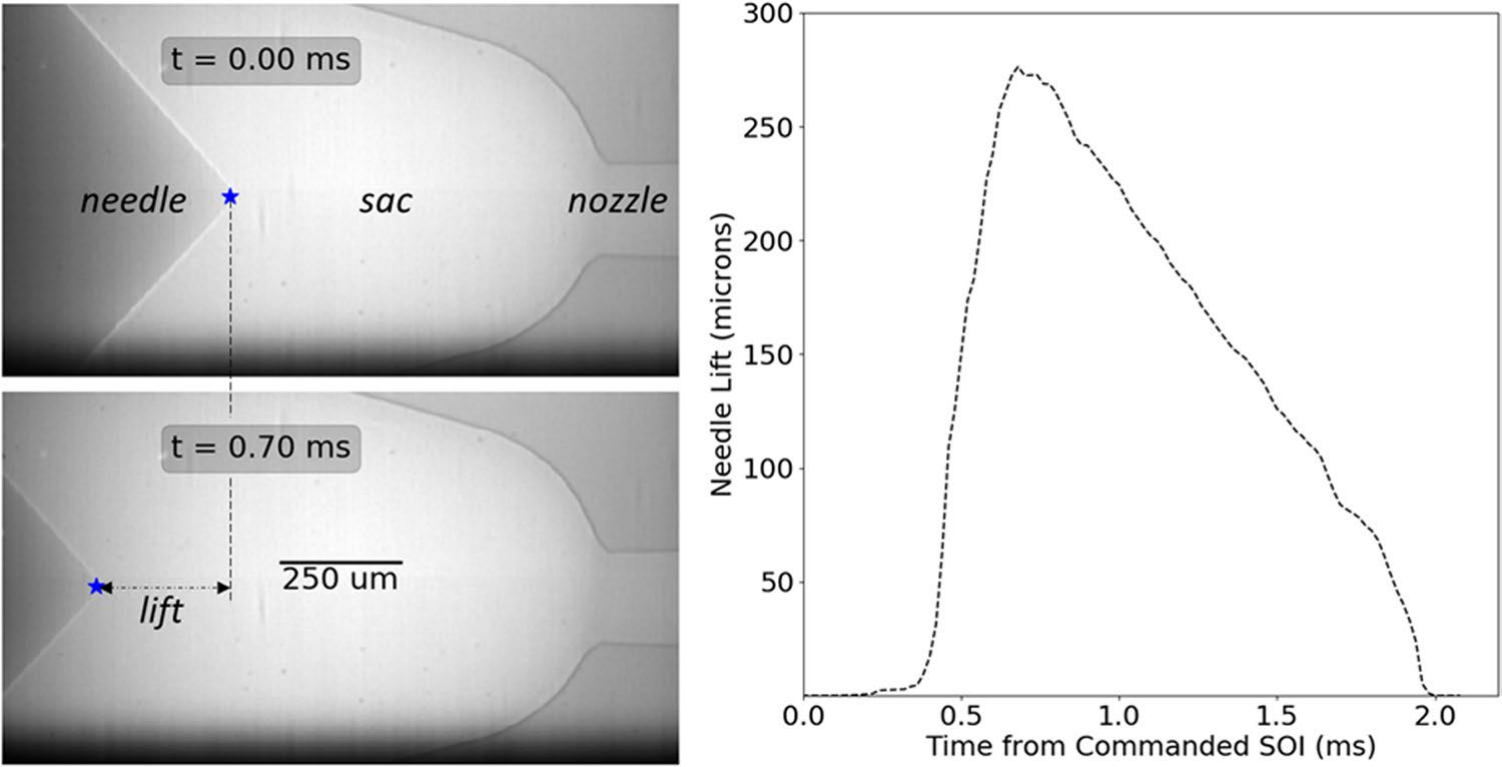
High-speed X-ray imaging of needle lift during fuel injection illustrates the timescale for subsequent flow imaging experiments. Time t = 0 ms is the electronically commanded start of injection (SOI).

The X-ray images in Fig. 2 are two frames from an acquisition of a 2 ms injection event at 50,000 fps. The resolution is 1.9*μ*m/pixel. The figure also shows the three main components in the diesel fuel injector – the needle, sac and nozzle. Before a fuel injection event commences, the needle is seated, preventing fuel from flowing into the sac. When the start of injection (SOI) is commanded with an electronic trigger, the needle lifts and creates a gap through which fuel floods the sac and subsequently flows through the orifice, or nozzle. At the end of injection, the needle lowers and closes the gap, thereby stopping the flow of fuel. In the sequence of images, the motion of the needle is tracked by implementing a normalized cross-correlation algorithm, and the trajectory along the nozzle axis (lift) is extracted as shown on the right in Fig. 2. Maximum needle lift occurs at 0.7 ms. For optimum performance of the tracking algorithm, the images are acquired with increased phase contrast, highlighting the metallic edges of the needle and nozzle walls. In-line phase contrast imaging is a technique in which phase interference of X-ray photons occurring in regions with sharp gradients in refractive index is exploited^24,25^ for better contrast. This is achieved by increasing the sample-to-detector distance (75 cm in these experiments).

### First attempt: 3D Snapshot at Maximum Needle Lift

**Figure 3:**
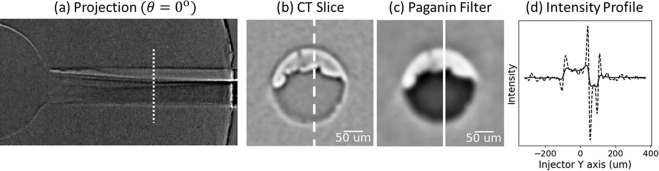
Illustration of the CT reconstruction process^21^ for one slice drawn from the projection images, shown in (**a**). Intensity profile is plotted (**d**) for the marked lines on the reconstructed slices CT Slice (**b**) and Paganin Filter (**c**) along the injector Y axis as shown.

As evident from the needle lift profile from Fig. 2, imaging the two-phase flow during the fuel injection event would demand at least microsecond time resolution to capture the transients during start and end of injection. This is extremely challenging, even with the maximum available flux at the synchrotron beamline (polychromatic or white beam), because the low-energy photons that are most sensitive to hydrocarbon fuels are absorbed by the steel body (see *Methods*). Hence, there are no perceptible features of the flow observed in the image from Fig. 2 at t = 0.7 ms, even though it was acquired during the middle of the injection event. As a first attempt to visualize the flow, the commanded injection duration was increased such that the needle was held at maximum needle lift for at least 1 ms. This allowed us to image at 1 ms exposure and capture a snapshot of the flow at maximum needle lift during the quasi-steady period from t = 1 to t = 2 ms. Next, the image acquired between t = −1 ms and t = 0 ms, i.e. before the commanded start of injection, was used to subtract the background field, creating a difference image that revealed strong flow separation occurring from the sharper side of the inlet to the nozzle. By averaging approximately 214 repeated injection events, the image in Fig. 3 (left) was obtained. The signal-to-noise ratio of these images is ~ 10, calculated as *μ*^2^/σ^2^. Next, the fuel injector was rotated in 0.5 degree steps and the acquisition was repeated for a total rotation of 180 degrees.

The images acquired by the procedure described above (2.1 *μ*m /pixel) were used as projections for reconstructing a 3D volume with a slice-by-slice Fourier transform reconstruction algorithm (“gridrec”) available in the open-source Python module TomoPy^26^. While rotating the fuel injector, it was observed that the nozzle precessed within the field of view by over 10 *μ*m, causing significant motion artifacts. To compensate for this misalignment, a normalized cross-correlation algorithm was employed to register the images based on the position of the needle tip upstream of the sac. An example reconstructed slice is shown in Fig. 3b. The interface between liquid and gas is quite sharp, showing distinct phase contrast. The fact that this interface is so sharp in the images suggests that this interface is quite stable during the middle of the injection and repeatable between injection events; if it were not, the variability in the interface would cause blur in the images.

**Figure 4:**
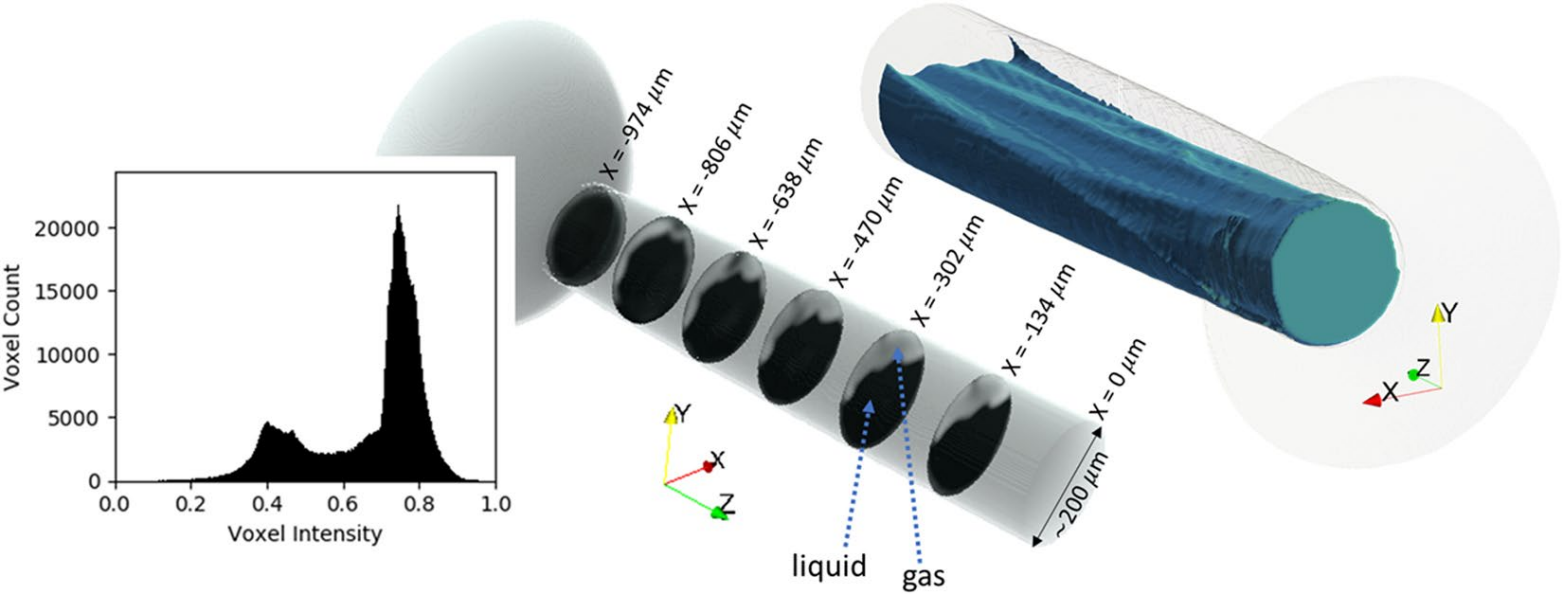
Slices from the fully reconstructed CT volume (left) show flow separation and cavitation leading up to the nozzle exit (nozzle length ~ 1  m). As per the coordinate system, x = 0 ***μ***m is at the nozzle tip. The inset figure shows histogram of voxel intensity in the slices, indicating a distinct binarizable intensity map, with high and low intensity corresponding to liquid and gas phases respectively. 3D volume rendering of the liquid phase is shown on the right (blue-green), further illustrating the wrinkled nature of the liquid-gas interface.

After reconstruction, a low-pass Paganin filter^26,27^ was applied to retrieve information from the in-line phase-contrast effects in the reconstructed slice. In Fig. 3(d), an intensity profile along the vertical axis shows the effect of the Paganin filter, with the intensity of the original image (b) shown as a dashed line, and the filtered image (c) as a solid line. The sharp phase contrast edges are smoothed without noticeable change in the overall intensity map. Although not quantitative with polychromatic X-rays, the Paganin filter makes the data binarizable, allowing the liquid-gas interface to be tracked. The darker pixels correspond to higher density material (liquid phase) and the lighter pixels correspond to the gas phase. Hence, it is possible to visualize the morphology of the two-phase flow using this information. The region outside the nozzle represents the noise floor since it is the residual between foreground and background frames. An iso-surface representing the walls of the nozzle was extracted from a separate reconstruction of the background frame (t < 0 ms). This iso-surface was used to clip voxels that do not belong inside the nozzle, resulting in Fig. 4 that shows six of the ~ 500 axial slices from the fully reconstructed CT volume, starting from the nozzle entrance and ending just upstream of the nozzle exit. Figure 4 also shows volume rendering of the liquid core, that helps visualize the wrinkled morphology of the liquid-gas interface. Some tessellation is observed in this rendering. This is because at 2.1*μ*m voxel size, a 200 *μ*m diameter flow cross-section is represented by less than 100 voxels.

The slices and volume rendering shown reveal the preferential formation of a gaseous layer that begins on the upper surface at the inlet of the orifice and extends to the exit. The onset location of the gaseous layer coincides with the inlet corner along +Y, which is known to have the sharpest radius of curvature^13^. Interestingly, the liquid-gas interface is wrinkled and may indicate the presence of a liquid jet (characterized by a higher density zone) penetrating halfway into the nozzle, enclosed by the gas layer. This jet could be highly transient and therefore shows weak contrast in this time-averaged snapshot through the steady flow part of the injection. Further investigation of this feature required an imaging study at higher frame rates, which is presented next.

### Time-resolved 3D imaging at 10 kHz

**Figure 5:**
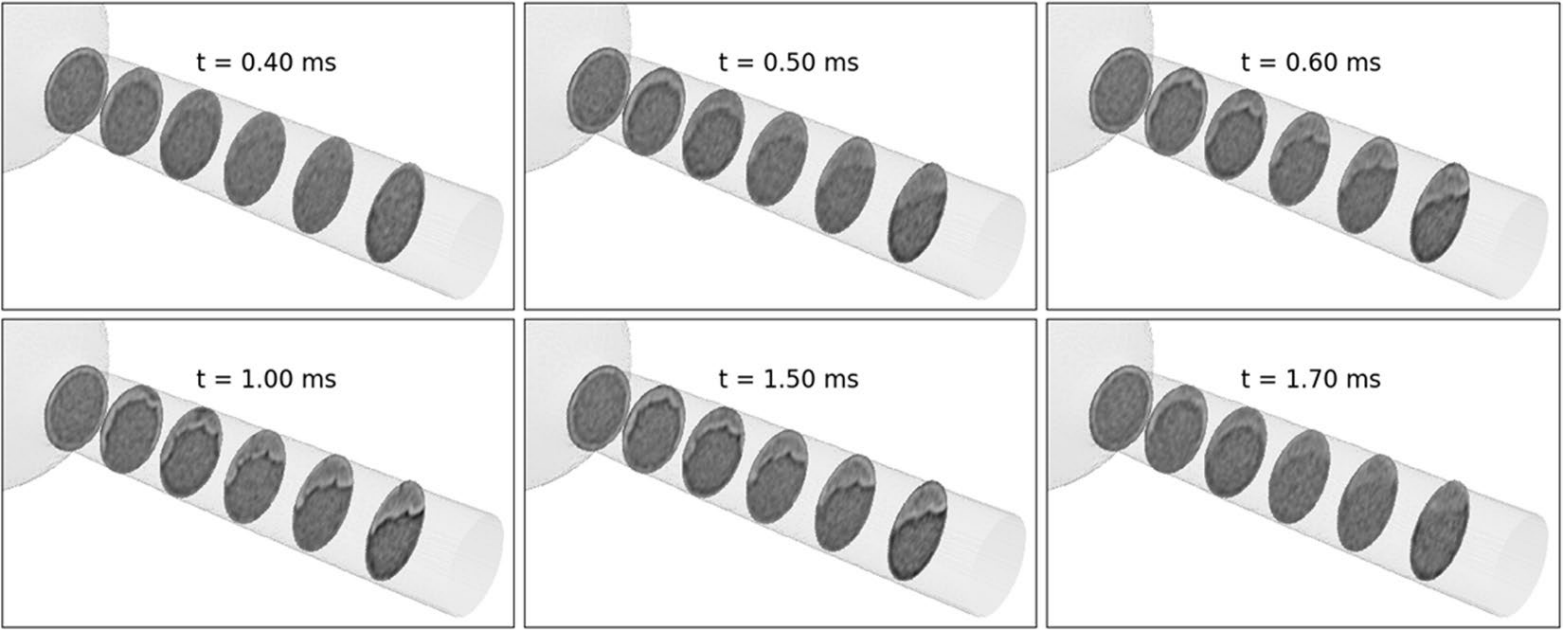
3D snapshots at various time-steps acquired at 100 ***μ***s time-resolution show the development of the flow as the needle lifts during the fuel injection. The coordinates for the slices are the same as in Fig. 4.

The results presented so far provide a unique visualization of two-phase effects inside a real fuel injector nozzle. To obtain these data, the needle lift profile was altered to allow a very long fuel injection and obtain a snapshot time-averaged over 1 ms. Hence, some questions remain unanswered, such as the time-scale for achieving fully developed flow, the potential appearance of a tiny liquid jet that penetrates through the gas layer, and the wrinkled nature of the liquid-gas interface. As a follow-up, the above campaign was repeated with the commanded injection and needle lift profile shown in Fig. 2. To capture the transient flow, the exposure time was reduced to 100 *μ*s (10,000 fps). The lower exposure time resulted in a much poorer signal-to-noise ratio, so each acquisition was averaged over 338 injection events (as opposed to 214 in the previous results). Based on the available time to complete the measurements, projections were acquired at an angular resolution of 1.875 degrees, which resulted in only 96 projections over a 180 deg rotation. Although more projections are preferred, this number is sufficient for the reconstruction algorithm because the total diameter of the flow cross-section being reconstructed is approximately 100 pixels (2.1 micrometer/pixel). Because these data showed poor absorption contrast (SNR ~ 1), the phase contrast was critical to visualizing the flow morphology. Therefore, the Paganin phase retrieval filter was not applied to the resulting reconstructions. Figure 5 shows 3D snapshots of the flow at select time steps, with the same time scale as in the needle lift profile from Fig. 2. The flow begins between t = 0.4 and 0.5 ms and is fully developed by t = 1.0 ms. As the needle starts to close, the flow recedes and stops after t = 1.7 ms. Notably, the frames before t = 0.6 ms and after 1.5 ms appear blurred. This is because the time-resolution of 0.1 ms was not sufficient to capture the start and end of injection transients, which occur at microsecond timescales as evident from the needle-lift profile from Fig. 2.

## Discussion

As mentioned before, the objective of this work was to investigate cavitation and flow separation in a practical diesel injector nozzle without any modifications for optical access and characterize the influence of surface features in steel orifices. To the best of our knowledge, our work is unique in that context and our observations are discussed below. Visualization inside a steel nozzle also introduced significant limitations to the imaging technique and noise statistics in the data. A discussion to that end is included in the *Methods* section.

**Figure 6:**
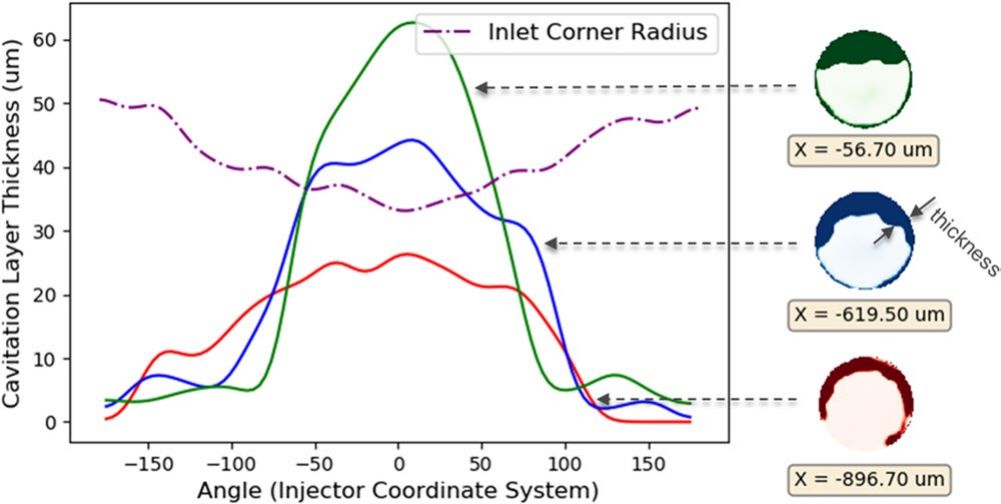
Cavitation/gas layer thickness at three axial distances along the nozzle, plotted against azimuthal angle, compared with local inlet corner radius^21^.

To correlate the dependence of flow-separation on nozzle geometry, the CT data from Fig. 4 was binarized by setting a threshold between the two prominent intensity distributions corresponding to gas and liquid phase. The boundary between the liquid and gas layer was then used to define a *cavitation layer thickness* as shown in Fig. 6. The thickness is plotted against the azimuthal angle θ with respect to the X axis (counter-clockwise is positive). The radius of curvature at the inlet corner for this nozzle was previously measured and adapted from Sforzo *et al*.^13^. There is good agreement between the extent of flow separation and the inlet corner radius. When the radius is smallest, the flow separation is largest. The micron-scale surface features at the inlet corner (shown in Fig. 1) cause variations in the measured inlet corner radius and appear to cause the *wrinkled effect* on the liquid-gas interface. Mitroglou *et al*. had previously visualized cavitation in a round polymer nozzle with similar asymmetric inlet corner radii and demonstrated the “crescent moon” shape of cavitation cross-section in a rounded nozzle as observed in this study^17^. However, the wrinkled shape of the cavitation layer in our data arises due to the rough surface of a steel nozzle and is resolved because of the high spatial resolution.

**Figure 7:**
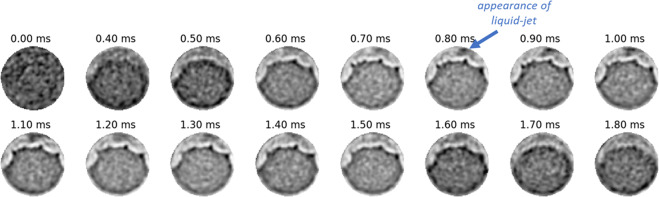
Slice of flow cross-section at x = −638 ***μ***m at various time-steps showing the emergence of liquid-jet in the gas layer.

Another interesting feature in the flow is the possibility of a liquid jet that passes through the gas layer and persists halfway down the length of the nozzle. This is seen in the slices from Fig. 4 between x = −974 *μ*m and x = −470 *μ*m. The origin of this liquid jet may be related to a prominent ridge at the nozzle inlet and is visible in the surface representation in Fig. 1 (right). The ridge is approximately 20 *μ*m wide. The axial slices at x = −638 *μ*m for various time-steps are shown in Fig. 7. The liquid jet appears between 0.8 and 0.9 ms after commanded SOI and disappears between 1.4 and 1.5 ms. The contrast for this feature is weaker and blurrier than the liquid phase in the core of the nozzle. This indicates that the feature is highly transient and a time-resolution of 10 kHz cannot accurately capture it’s evolution. Regardless, it is certainly a repeatable feature in all injections since it appears in these statistically averaged snapshots from several thousand injections.

A significant effect of cavitation in this nozzle is flow blockage – i.e. reduction in the effective flow area. By counting the pixels belonging to the liquid and gas phases in the binarized data, we estimate the effective flow area to be between 60–70% of the total cross-sectional area of the orifice. It is important to note here that our experimental technique and analysis are not sufficient for accurately calculating liquid volume fraction distribution. While the histogram of the data (Fig. 4) shows two prominent phases, it is likely that liquid parcels are present in the gas layer while dissolved gas is present in the liquid. Regardless, our observations explain the findings of Payri *et al*.^28^, who measured the mass flow rate of fuel (rate of injection) through Spray C and another nozzle (Spray D) under similar operating conditions as this study. They found that despite the larger diameter of the Spray C nozzle as compared to Spray D, the rate of injection was found to be lower in Spray C. Furthermore, Spray C showed a larger vapor spreading angle possibly due to enhanced air entrainment^29,30^. It is possible that cavitating flow promoted enhanced fuel-air mixing in the near-nozzle region.

As a final comment, this work presents a first-time 3D visualization of internal flow inside a practical steel nozzle, through approximately 2 mm of steel, with 2 *μ*m pixel resolution. Although these data only captures effects that are repeatable across injection events, they provide unique insights on the effect of manufacturing variabilities in nozzle geometry on the morphology of bounded flows. We have provided a data set for computational fluid dynamics simulations that seek to accurately predict multiphase effects occurring over realistic surfaces at micron-scales.

## Methods

### Experimental Setup

**Figure 8:**
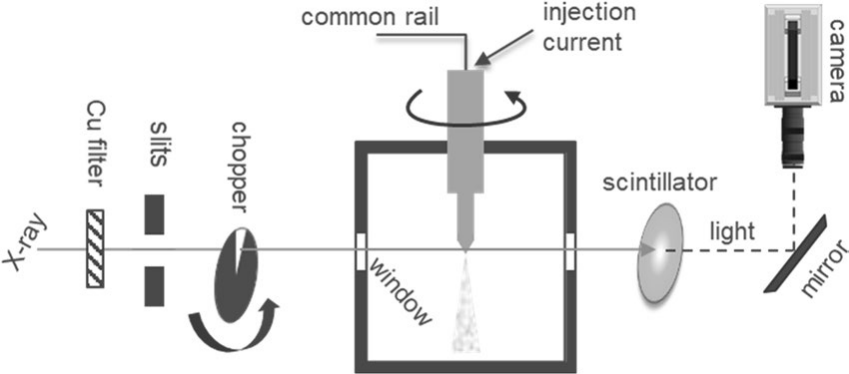
Schematic of experiment illustrating high-speed X-ray imaging setup^21,22^.

The imaging experiments were conducted at the 7-BM^31^ and 32-ID^32^ beamlines of the Advanced Photon Source at Argonne National Laboratory. The experimental technique is based on previous efforts towards internal flow and cavitation imaging^12,13^. In this work, the technique was extended to image the internal flow in the nozzle from several hundred viewing angles. The schematic in Fig. 8 illustrates the experimental setup. The fuel injector was mounted in a chamber equipped with a rotational stage such that the fuel injector rotates inside the chamber. A common rail fuel injection system supplied high pressure fuel (99% pure n-dodecane) at 150 MPa to the injector. The injector was triggered using an electronic driver with commanded duration of 480 *μ*s to acquire the needle lift profile (Fig. 2) and 10 kHz snapshots (Fig. 5). As discussed previously, the commanded duration was increased to 2 ms for acquiring the steady-flow snapshot with 1 ms exposure time (Fig. 4). The injection command was repeated at 3 Hz for internal flow imaging experiments and 1 Hz for needle lift characterization. To clear fuel vapor from the ambient gas, the chamber was purged with nitrogen gas, refreshing at 6 liters per minute. The pressure and temperature of the fuel injector and chamber were monitored to be consistent with the operating conditions presented in Table 1. The spray chamber was fitted with X-ray-transparent Kapton windows and was mounted on linear translation stages to control the field-of-view.

**Table 1:** Summary of experimental conditions.

Injection pressure	150 MPa
Ambient pressure	0.1 MPa
Fuel temperature	25 deg C
Ambient temperature	25 deg C

The imaging apparatus shown in Fig. 8 represents the flow imaging campaign at 7-BM; the imaging of needle lift at 32-ID beamline follows a similar setup which has been described previously by the authors^33,34^. A polychromatic X-ray beam generated by a bending magnet^31^ was passed through a 250 *μ*m thick copper filter. The beam shape was conditioned with a set of X-ray slits, and a mechanical beam chopper was used to reduce heat load on the sample. The beam transmitted through the sample (injector nozzle) was converted to visible light when incident on a 500 *μ*m thick yttrium aluminum garnet scintillator. The visible light was then imaged by a 5X objective lens onto a high-speed CMOS detector (Photron FastCam SA-4) resulting in a 2 *μ*m/pixel resolution.

### Imaging Technique

X-ray photons transmitted through the steel and liquid-gas flow interface are not only attenuated due to absorption but also redirected due to phase interference occurring in regions containing sharp gradients in refractive index, such as metal boundaries or a liquid-gas interface^24,25^. This added contrast due to refraction effects (so-called “phase contrast”) complements the relatively weak absorption signal and allows better visualization of the morphology of the two-phase flow. Phase contrast can be increased by optimizing the sample-to-detector distance and was set to 75 cm in our experiments. The use of polychromatic or *white beam* severely limits the extraction of quantitative density-field data from the combination of absorption and phase-contrast effects, because they are both dependent on photon energy. However, considering the unique challenge of imaging inside a 1 mm thick injector tip, it is critical to use all the available flux.

The image formed on the CMOS camera picks up noise from several sources. These include photon statistics of the X-ray beam and the scintillator emission, fixed pattern noise due to sensitivity variations in the CMOS array, and electrical noise. To appreciate the trade-off between nozzle material, time-resolution and signal-to-noise ratio, we can consider the X-ray photon statistics in a path consisting of 2 mm of steel, 0.170 mm of liquid fuel and 0.030 mm of gas/vapor. The incident X-ray beam from the 7-BM bending magnet has a total flux of ~ 10^15^ photons/s/mm^2^ integrated across photon energies 1–100 keV^31^. The beam first passes through a 250 *μ*m copper filter to remove low-energy photons which would otherwise be absorbed by the steel and consequently heat load the injector. The effective beam energy is approximately 50 keV. After passing through the steel path, the effective beam energy is over 80 keV and more than 98.5% of the flux has been absorbed, leaving about 4 × 10^12^ ph/s/mm^2^ to be absorbed in the 0.17 mm path of liquid fuel. The overall transmission of these photons through the liquid fuel path integrated over the energy distribution is over 99.97%. After background subtraction, one may expect less than one photon to be incident on one pixel (4 *μ*m^2^) per ms of exposure. This is highly impractical and hence we imaged over 214 injection events to acquire an ensemble-averaged snapshot at each projection angle for the tomography dataset. At 214 repetitions for the 1 ms exposure, we can expect a signal-to-noise ratio of 32 assuming only Poisson statistics as the source of error. To acquire the 10 kHz time-resolved data, we averaged over 338 events, expecting a signal-to-noise ratio of 12 in this case.

## References

[1] Matusik KE (2018). High-resolution X-ray tomography of Engine Combustion Network diesel injectors. Int. J. Eng. Res.

[2] Mitroglou N, Stamboliyski V, Karathanassis IK, Nikas KS, Gavaises M (2017). Cloud cavitation vortex shedding inside an injector nozzle. Experimental Thermal and Fluid Science.

[3] Arcoumanis, C., Flora, H., Gavaises, M. & Badami, M. Cavitation in Real-Size Multi-Hole Diesel Injector Nozzles. in *SAE Technical Paper Series* **1**, (SAE International, 2000).

[4] Gavaises, M., Papoulias, D., Andriotis, A., Giannadakis, E. & Theodorakakos, A. Link between cavitation development and erosion damage in diesel injector nozzles. in *SAE Technical Paper Series* **1**, (SAE International, 2007).

[5] Magnotti, G. M., Battistoni, M., Saha, K. & Som, S. In *Proceedings of the 10th international symposium on cavitation (CAV2018)*10.1115/1.861851_ch87 (ASME Press, 2018).

[6] Gavaises M, Andriotis A, Papoulias D, Mitroglou N, Theodorakakos A (2009). Characterization of string cavitation in large-scale Diesel nozzles with tapered holes. Phys. Fluids.

[7] Blessing, M., König, G., Krüger, C., Michels, U. & Schwarz, V. Analysis of flow and cavitation phenomena in diesel injection nozzles and its effects on spray and mixture formation. in *SAE Technical Paper Series* **1**, (SAE International, 2003).

[8] Sou, A., Minami, S., Prasetya, R. & Pratama, R. H. X-Ray Visualization of Cavitation in Nozzles with Various Sizes. in *Proceedings of ICLASS 2015* at https://www.researchgate.net/publication/281459601_X-Ray_Visualization_of_Cavitation_in_Nozzles_with_Various_Sizes (ILASS, 2015).

[9] Arcoumanis C, Gavaises M, Flora H, Roth H (2001). Visualisation of cavitation in diesel engine injectors. Mécanique & Industries.

[10] Arcoumanis C, Gavaises M (1998). Linking nozzle flow with spray characteristics in a diesel fuel injection system. Atomiz. Spr..

[11] Payri R, Molina S, Salvador FJ, Gimeno J (2004). A study of the relation between nozzle geometry, internal flow and sprays characteristics in diesel fuel injection systems. KSME International Journal.

[12] Duke D (2014). X-ray Imaging of Cavitation in Diesel Injectors. SAE Int. J. Engines.

[13] Sforzo, B. A. *et al*. In *Proceedings of the 10th international symposium on cavitation (CAV2018)*10.1115/1.861851_ch90 (ASME Press, 2018).

[14] Mäkiharju SA (2013). Time-resolved two-dimensional X-ray densitometry of a two-phase flow downstream of a ventilated cavity. Exp. Fluids.

[15] Moon S, Huang W, Wang J (2019). First observation and characterization of vortex flow in steel micronozzles for high-pressure diesel injection. Experimental Thermal and Fluid Science.

[16] Karathanassis IK (2018). High-speed visualization of vortical cavitation using synchrotron radiation. J. Fluid Mech..

[17] Mitroglou N, Lorenzi M, Santini M, Gavaises M (2016). Application of X-ray micro-computed tomography on high-speed cavitating diesel fuel flows. Exp. Fluids.

[18] Bauer D, Barthel F, Hampel U (2018). High-speed x-ray CT imaging of a strongly cavitating nozzle flow. J. Phys. Commun..

[19] Bauer D, Chaves H, Arcoumanis C (2012). Measurements of void fraction distribution in cavitating pipe flow using x-ray CT. Meas. Sci. Technol..

[20] Engine Combustion Network | Engine Combustion Network Website. at https://ecn.sandia.gov/

[21] Tekawade, A., Sforzo, B. A., Matusik, K. E., Kastengren, A. L. & Powell, C. F. 3D Imaging of Cavitating Flow in a Diesel Injector at Practical Conditions using X-ray micro-CT. In at http://www.ilass.org/2/conferencepapers/63_2019.pdf (ILASS, 2019).

[22] Tekawade, A. *et al*. A comparison between CFD and 3D X-ray Diagnostics of Internal Flow in a CavitatingDiesel Injector Nozzle. In at http://www.ilass.org/2/conferencepapers/65_2019.pdf (ILASS, 2019).

[23] Tekawade, A., Sforzo, B. A., Matusik, K. E., Kastengren, A. L. & Powell, C. F. Application of synchrotron x-ray imaging and micro-CT to 4D visualization of multiphase flow inside a steel fuel nozzle (Conference Presentation). in *Developments in X-Ray Tomography XII* (eds. Müller, B. & Wang, G.) 33 10.1117/12.2527585 (SPIE, 2019).

[24] Kastengren A, Powell CF (2014). Synchrotron X-ray techniques for fluid dynamics. Exp. Fluids.

[25] Lee JS, Weon BM, Je JH (2013). X-ray phase-contrast imaging of dynamics of complex fluids. J. Phys. D, Appl. Phys..

[26] Gürsoy D, De Carlo F, Xiao X, Jacobsen C (2014). TomoPy: a framework for the analysis of synchrotron tomographic data. J Synchrotron Radiat.

[27] Paganin D, Mayo SC, Gureyev TE, Miller PR, Wilkins SW (2002). Simultaneous phase and amplitude extraction from a single defocused image of a homogeneous object. J. Microsc..

[28] Payri R, Gimeno J, Cuisano J, Arco J (2016). Hydraulic characterization of diesel engine single-hole injectors. Fuel.

[29] Gimeno J, Bracho G, Martí-Aldaraví P, Peraza JE (2016). Experimental study of the injection conditions influence over n-dodecane and diesel sprays with two ECN single-hole nozzles. Part I: Inert atmosphere. Energy Conversion and Management.

[30] Payri R, Salvador FJ, Gimeno J, Peraza JE (2016). Experimental study of the injection conditions influence over n-dodecane and diesel sprays with two ECN single-hole nozzles. Part II: Reactive atmosphere. Energy Conversion and Management.

[31] Kastengren A (2012). The 7BM beamline at the APS: a facility for time-resolved fluid dynamics measurements. J Synchrotron Radiat.

[32] Shen Q (2007). Dedicated full-field X-ray imaging beamline at Advanced Photon Source. Nuclear Instruments and Methods in Physics Research Section A: Accelerators, Spectrometers, Detectors and Associated Equipment.

[33] Viera JP (2016). Linking instantaneous rate of injection to X-ray needle lift measurements for a direct-acting piezoelectric injector. Energy Conversion and Management.

[34] Powell, C. F., Kastengren, A. L., Liu, Z. & Fezzaa, K. The effects of diesel injector needle motion on spray structure. *J. Eng. Gas Turbines Power* **133**, (2010).

